# Adherence to preoperative hand hygiene and sterile gowning technique among consultant surgeons, surgical residents, and nurses: a pilot study at an academic medical center in Indonesia

**DOI:** 10.1186/s13037-019-0193-5

**Published:** 2019-03-11

**Authors:** Adeodatus Yuda Handaya, Victor Agastya Pramudya Werdana

**Affiliations:** Digestive Surgery Division, Department of Surgery, Faculty of Medicine, Public Health and Nursing, Universitas Gadjah Mada/Dr. Sardjito Hospital, Jl. Kesehatan No. 1, Yogyakarta, 55281 Indonesia

**Keywords:** Gowning technique, Hand hygiene, Donning the gloves, Abdominal surgery

## Abstract

**Background:**

Healthcare-associated infections (HAI) is a major problem for patient safety and surgical site infection (SSI) is a type of HAI and the most common form of infection related to surgical health care. Transmission of microorganisms can be minimized by aseptic procedures. The main objective of this study is to compare adherence to preoperative sterile gowning and hand hygiene technique among consultant surgeons, surgical residents, and nurses.

**Methods:**

This research was conducted by observing the implementation of the pre-operative sterile gowning and hand hygiene technique of abdominal surgery by consultant surgeons, surgical residents, and nurses using aseptic instrument tests of the Objective Structured Clinical Examination (OSCE) Faculty of Medicine Universitas Gadjah Mada from August 10, 2018 to September 10, 2018. Observations were made when participants performed hand scrubbing, gowning, and donning the gloves procedures. The observer completed mobile online forms, so that the medical personnel under observation did not know that they were being observed.

**Results:**

Twelve consultant surgeons, 16 surgical residents, and 12 nurses were observed. All of the medical personnel showed a good score with total percentage mean 83.58%. The highest total mean score was achieved by consultant surgeons (86.39%), but mean score did not vary significantly between medical personnel (*p* = .091). In the hand scrubbing procedure, scrub the nail and palm using brush side and the skin of hand and arm using sponge side, in both hands had the lowest mean score (1.82 ± 1.152 of the maximum score of 4). While in the gowning procedure, taking and unfolding the sterile gown had the lowest mean score (1.97 ± .158 of the maximum score of 2). In the donning the glove procedure, grasping left glove with right hand and putting the glove over the left hand in opposite direction procedure had the lowest mean score (1.97 ± .158 of the maximum score of 2).

**Conclusions:**

The mean score of each group of health personnel in each section showed good results. Comparison of hand hygiene and gowning procedure performance between groups of health personnel did not show significant differences. However, larger scale research is needed after this pilot study.

## Background

Healthcare-associated infections (HAI) is a major problem for patient safety and lead to other problems such as increased financial burden, emotional stress, antibiotic resistance, and mortality. Surgical site infection (SSI) is a type of HAI and the most common form of infection related to surgical health care. SSI accounts for 14–16% of infections and occurs in about 5% of surgical patients [1–3].

Transmission of microorganisms can be minimized by the aseptic technique. Hand washing techniques in pre-operative aseptic procedures consist of several components such as initial hand washing, method of rubbing, drying of hands, wearing an operating gown, and wearing sterile gloves. One of the factors that influence the success of hand washing is the level of compliance with the recommended standard procedures [4–6]. The level of compliance with handwashing by workers in various health centers shows different results. Comparison between doctors and nurses health personnel in adhering to handwashing procedures also varies [4, 7].

The main objective of this study is to compare performance of consultant surgeons, surgical residents, and nurses in pre-operative aseptic procedures in the hand scrubbing, gowning, and donning the gloves procedures.

## Methods

### Design and study population

This research was conducted by observing the implementation of the pre-operative aseptic procedures of digestive surgery by consultant surgeons, surgical resident, and nurses using aseptic instrument tests of the Objective Structured Clinical Examination (OSCE) Faculty of Medicine Universitas Gadjah Mada. Dr. Sardjito General Hospital is a University Teaching Hospital and serves as a tertiary referral center.

This research was an observational descriptive cross-sectional study conducted from August 10, 2018 to September 10, 2018. The study population in this study were health personnel consisting of consultant surgeons, surgical residents, and nurses involved in digestive surgery in Dr. Sardjito General Hospital. This study included all consultant surgeons, surgical residents, and nurses who performed elective digestive surgery in a predetermined period of time. We excluded all consultant surgeons, surgical residents, and nurses who performed digestive surgery in emergency setting.

### Data collection

Data collection is done by consecutive sampling, covert direct observations were made by one surgical resident (MD) when participants performed hand scrubbing, donning the gown, and donning the gloves in the preoperative setting. Observers already know the steps of the preoperative aseptic procedure and have been trained in using the research form. The observer completed mobile online forms through a smartphone, so that the health personnel under observation did not know that they were being observed.

### Detailed procedures observed

In the method of scrubbing & drying hand, there are 13 steps observed (Table 1). The maximum score that can be obtained in this section is 34. Procedure 2,3,5,6 in the method of scrubbing and drying hand has a maximum score of 4, the other procedure has a maximum score of 2.

**Table 1:** Detailed procedure observed in the method of scrubbing & drying hand

Method of scrubbing & drying hand	
1. Taking the sufficient amount of liquid soap into sterile brush	
2. Doing scrub procedure evenly by hand to proximal elbow (2 min for each of hands) using scrubbing, done for both side (left and right) and rinsing by rotation	
3. Scrub the nail and palm using brush side and the skin of hand and arm using sponge side, in both hands.	
4. Discarding the brush carefully to the disposal bin	
5. Doing scrub procedure evenly by hand to half of distal lower arm (1 min for each arm) just using soap, done for both side and rinsing by rotation	
6. Doing scrub procedure evenly by hand to half of proximal lower arm (2 min for each of arms) just using soap, done for both side and rinsing by rotation	
7. Turning off the water flow by using elbow or pedal (without using hand)	
8. Then, move to enter the operation room through “butterfly” door by step backward (without touching the door)	
9. During the entire steps of surgical hand disinfection, hands should be keep slightly higher than lower arms, both hands should be on visual fields. The hand and lower arms must not touch the unclean surface. Hold on this position until the gloves are worn.	
10. Taking the sterile towel and dividing it into two parts	
11. Drying the hands one by one, sequentially from distal to proximal area	
12. The hand must be nested in a towel while its drying another hand to protect it from distal to proximal contact of both hands	
13. Discarding the towel directly to the basin	

In the donning the gowning procedure, there are 3 steps observed (Table 2). The maximum score that can be obtained in this section is 6, each step has a maximum score of 2.

**Table 2:** Detailed procedure observed in donning the gown procedures

Donning the gown	
1. Taking the sterile gown by grasping the inner part of the gown especially to choose the neck part	
2. Unfolding it away from the body. Avoiding contact to the floor	
3. Donning the gown by hands and arms are extending through the long sleeves of the gown, only until the cuffs level (for using closed method of gloving)	

In the donning the glove procedure, there are 3 steps observed (Table 3). The maximum score that can be obtained in this section is 16, each step has a maximum score of 2.

**Table 3:** Detailed procedure observed in the donning the glove

Donning the gown	
1. Opening the wrapper of the gloves by hands inside the sleeves of the gown	
2. Inside the sleeve, the left hand is opened and facing up	
3. Grasping left glove with right hand (closed by the sleeve) and put the glove over the left hand in opposite direction	
4. Grasping the edge of the glove with both hands and making the broad weep motion simultaneously to insert the left hand inside the glove. While inserting into the glove, the left fingers are in adduction then simultaneously abducted.	
5. The right hand pulls the edge of the glove and the edge of the sleeve, the glove should cover the cuff part of the sleeve	
6. Repeating the same procedure toward the right hand	
7. Adjusting the position of the gloves	
8. During this procedure, hands DO NOT TOUCH the outer parts of the gloves	

### Statistical analysis

We analyzed the mean of total score of each section among the health personnel. One way Anova tests were used to compare the mean score data. IBM SPSS Statistics version 23 (SPSS Chicago, IL, USA) was used for statistical analysis. We also present the mean score for each detailed procedure among the health personnel.

## Results

We observed 40 health personnel consisting of 12 consultant surgeons, 16 surgical residents, and 12 nurses who performed surgery in Dr. Sardjito Hospital Yogyakarta. We divided the aseptic procedures into three categories: method of scrubbing and drying the hands, donning the gown, and donning the gloves. We analyzed the frequency and average scores per group (Table 4).

**Table 4:** Number of samples observed

Medical personnel	N	(%)
Consultant Surgeons	12	30.0
Surgical Residents	16	40.0
Nurses	12	30.0
Total	40	100.0

### Method of scrubbing & drying hand

**Fig. 1:**
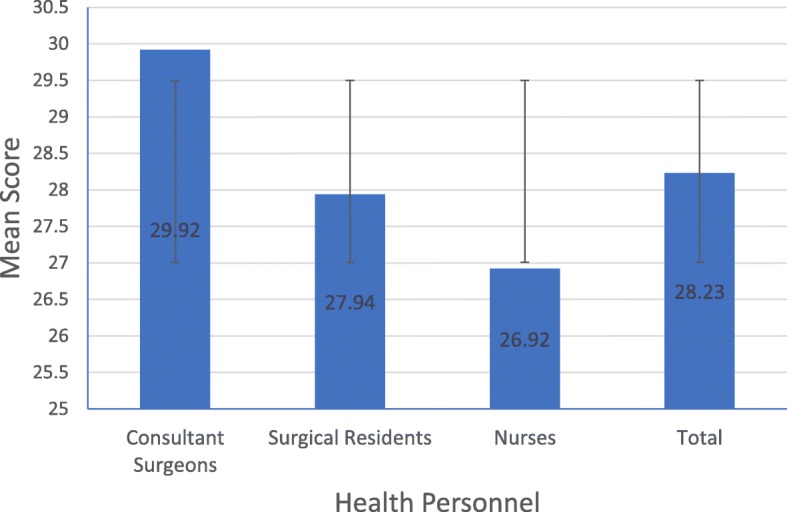
Mean score of the method of scrubbing & drying hand

In the 40 observed health personnel, the highest score of 34 was reached by four consultant surgeons (10% of total), while the lowest score with a value of 20 was reached by one resident doctor (2.5% of total). The maximum score that can be achieved in this section is 34. Consultant surgeons had the highest average scores (29.92 ± 3.655), while nurses had the lowest average score (26.92 ± 2.539). The mean score of the method of scrubbing and drying hand did not vary significantly between health personnel (*p* = .060) (Fig. 1 and Table 5).

**Table 5:** Mean score of the method of scrubbing & drying hand

	N	Mean ± SD	Minimum	Maximum
Consultant Surgeons	12	29.92 ± 3.655	22	34
Surgical Residents	16	27.94 ± 2.886	20	31
Nurses	12	26.92 ± 2.539	22	30
Total	40	28.23 ± 3.198	20	34

### Donning the gown

**Fig. 2:**
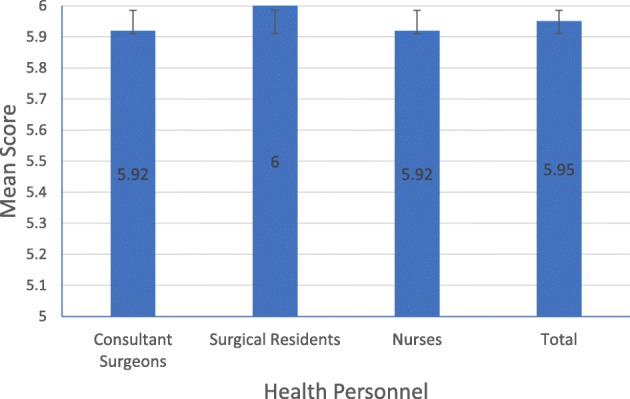
Mean score in the donning the gown procedure

All of the health personnel categories reached the maximum score, with the highest number of surgical residents with 16 personnel (40% of total). The maximum score that can be achieved in this section is 6. Consultant surgeons and nurses had one health personnel each (2.5% of total, each) with a score of 5, the lowest score. Surgical residents had the highest average scores (6.00 ± 0.000), while both consultant surgeons and nurses had the lowest average score (5.92 ± 0.289). The mean score of method of donning the gown did not vary significantly between health personnel (*p* = .516) (Fig. 2 and Table 6).

**Table 6:** Mean score in the donning the gown procedure

	N	Mean ± SD	Minimum	Maximum
Consultant Surgeons	12	5.92 ± .289	5	6
Surgical Residents	16	6.00 ± .000	6	6
Nurses	12	5.92 ± .289	5	6
Total	40	5.95 ± .221	5	6

### Donning the gloves

**Fig. 3:**
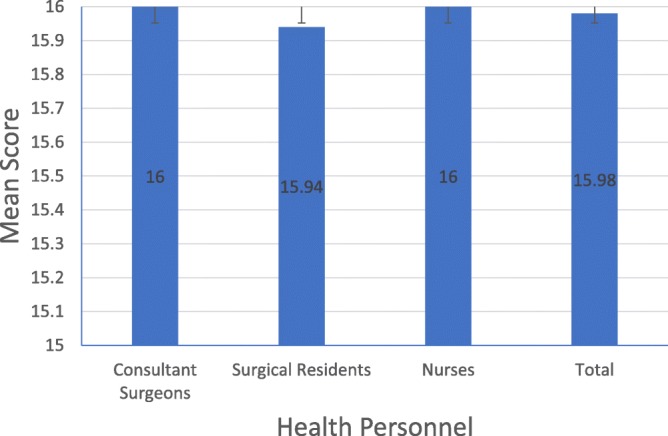
Score in the donning the glove procedure

All of the health personnel observed from consultant surgeons and nurses categories reached the maximum score (16), while one of the surgical residents had the lowest score (15). The maximum score that can be achieved in this section is 16. Both consultant surgeons and nurses had the highest average scores (16.00 ± 0.000), while surgical residents had the lowest average score (15.94 ± 0.250). The mean score of the method of donning the gloves did not vary significantly between health personnel (*p* = .484) (Fig. 3 and Table 7).

**Table 7:** Mean score of the donning the glove procedure

	N	Mean ± SD	Minimum	Maximum
Consultant Surgeons	12	16.00 ± .000	16	16
Surgical Residents	16	15.94 ± .250	15	16
Nurses	12	16.00 ± .000	16	16
Total	40	15.98 ± .158	15	16

### Total score

**Fig. 4:**
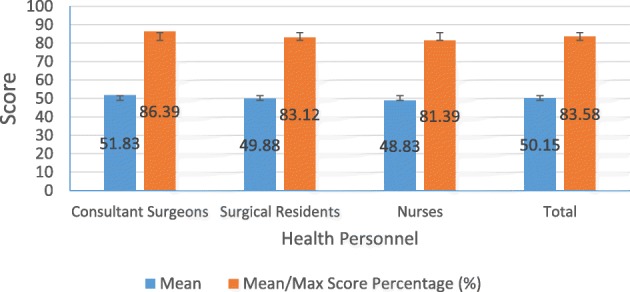
Total score and percentage of maximum score

From the three groups of health personnel, consultant surgeons had the highest average scores (51.83 ± 3.664), followed by surgical residents (49.93 ± 2.849), and nurses (48.91 ± 2.548). The average score of the three groups of health personnel is 50.15 ± 3.167. With a maximum total score of 60, the average percentage of each group was: consultant surgeons 86.39%, surgical residents 83.12%, and nurses 81.39%, with an overall average of 83.58%. The mean score of total procedures did not vary significantly between health personnel (*p* = .057) (Fig. 4 and Tables 8, 9, 10).

**Table 8:** Total score of three parts procedure

	N	Mean ± SD	Minimum	Maximum
Consultant Surgeons	12	51.83 ± 3.664	44	56
Surgical Residents	16	49.88 ± 2.849	42	53
Nurses	12	48.83 ± 2.443	44	52
Total	40	50.15 ± 3.167	42	56

**Table 9:** Percentage score with maximum score

	N	Mean/Max Score Percentage (%) ±	Minimum	Maximum
Consultant Surgeons	12	86.39 ± 6.106	73.33	93.33
Surgical Residents	16	83.12 ± 4.748	70.00	88.33
Nurses	12	81.39 ± 4.072	73.33	86.67
Total	40	83.58 ± 5.277	70.00	93.33

**Table 10:** Mean score of detailed procedure. Procedure 2,3,5,6 in the method of scrubbing and drying hand has maximum score of 4, the other procedure has maximum score of 2

Detailed procedure	Medical personnel							
	Surgeons		Surgical residents		Nurses		Total	
	Mean	Std. Deviation	Mean	Std. Deviation	Mean	Std. Deviation	Mean	Std. Deviation
Method of Scrubbing & Drying Hand								
1. Taking the sufficient amount of liquid soap into sterile brush	1.67	.492	1.50	.516	1.75	.452	1.62	.490
2. Doing scrub procedure evenly by hand to proximal elbow (2 min for each of hands) using scrubbing, done for both side (left and right) and rinsing by rotation	3.67	.492	3.38	.619	3.17	.577	3.40	.591
3. Scrub the nail and palm using brush side and the skin of hand and arm using sponge side, in both hands.	2.33	1.435	1.62	1.025	1.58	.900	1.82	1.152
4. Discarding the brush carefully to the disposal bin	1.58	.515	1.38	.500	1.50	.522	1.47	.506
5. Doing scrub procedure evenly by hand to half of distal lower arm (1 min for each arm) just using soap, done for both side and rinsing by rotation	3,67	.492	3.25	.683	3.00	.853	3.30	.723
6. Doing scrub procedure evenly by hand to half of proximal lower arm (2 min for each of arms) just using soap, done for both side and rinsing by rotation	3.67	.492	3.38	.619	3.08	.669	3.38	.628
7. Turning off the water flow by using elbow or pedal (without using hand)	2.00	.000	2.00	.000	2.00	.000	2.00	.000
8. Then, move to enter the operation room through “butterfly” door by step backward (without touching the door)	2.00	.000	1.94	.250	2.00	.000	1.98	.158
9. During the entire steps of surgical hand disinfection, hands should be keep slightly higher than lower arms, both hands should be on visual fields. The hand and lower arms must not touch the unclean surface. Hold on this position until the gloves are worn.	2.00	.000	2.00	.000	2.00	.000	2.00	.000
10. Taking the sterile towel and dividing it into two parts	1.92	.289	1.87	.342	1.75	.452	1.85	.362
11. Drying the hands one by one, sequentially from distal to proximal area	1.83	.389	1.94	.250	1.75	.452	1.85	.362
12. The hand must be nested in a towel while its drying another hand to protect it from distal to proximal contact of both hands	1.92	.289	1.94	.250	1.83	.389	1.90	.304
13. Discarding the towel directly to the basin	1.667	.4924	1.750	.4472	1.500	.5222	1.650	.4830
Donning the Gown								
1. Taking the sterile gown by grasping the inner part of the gown especially to choose the neck part	1.92	.289	2.00	.000	2.00	.000	1.97	.158
2. Unfolding it away from the body. Avoiding contact to the floor	2.00	.000	2.00	.000	1.92	.289	1.97	.158
3. Donning the gown by hands and arms are extending through the long sleeves of the gown, only until the cuffs level (for using closed method of gloving)	2.00	.000	2.00	.000	2.00	.000	2.00	.000
Donning the Glove								
1. Opening the wrapper of the gloves by hands inside the sleeves of the gown	2.00	.000	2.00	.000	2.00	.000	2.00	.000
2. Inside the sleeve, the left hand is opened and facing up	2.00	.000	2.00	.000	2.00	.000	2.00	.000
3. Grasping left glove with right hand (closed by the sleeve) and put the glove over the left hand in opposite direction	2.00	.000	1.94	.250	2.00	.000	1.97	.158
4. Grasping the edge of the glove with both hands and making the broad weep motion simultaneously to insert the left hand inside the glove. While inserting into the glove, the left fingers are in adduction then simultaneously abducted.	2.00	.000	2.00	.000	2.00	.000	2.00	.000
5. The right hand pulls the edge of the glove and the edge of the sleeve, the glove should cover the cuff part of the sleeve	2.00	.000	2.00	.000	2.00	.000	2.00	.000
6. Repeating the same procedure toward the right hand	2.00	.000	2.00	.000	2.00	.000	2.00	.000
7. Adjusting the position of the gloves	2.00	.000	2.00	.000	2.00	.000	2.00	.000
8. During this procedure, hands DO NOT TOUCH the outer parts of the gloves	2.00	.000	2.00	.000	2.00	.000	2.00	.000

## Discussion

In this study, we did a covert direct observational study to observe the adherence of hand hygiene and gowning technique which is the part of the pre-operative aseptic procedure. Observational method is considered as the standard method for research on hand hygiene adherence [3, 8].

Among the three groups of health personnel, consultant surgeons had the highest average scores (51.83 ± 3.664), followed by surgical residents (49.93 ± 2.849), and nurses (48.91 ± 2.548). The average score of the three groups of health personnel was 50.15 ± 3.167. The three groups of health personnel reached a good score. With a maximum total score of 60, the average percentage of each group was: consultant surgeons 86.39%, surgical residents 83.12%, and nurses 81.39%, with an overall average of 83.58%. Mean score of total procedures did not vary significantly between health personnel (*p* = .057).

These results indicate good compliance by the three groups of health personnel. Since the Sardjito Hospital is a national referral hospital, implementing all actions according to standard operational procedures is very important. Study by Krediet et al. showed that hand hygiene practices in the operating theatre is low in one of the academic hospital in Netherland. However, there are several differences in the scoring system used and the actions observed [9]. Several studies regarding the prevention of infection and compliance with hand washing or aseptic techniques among health personnel have shown different results. Research of hand hygiene and glove use by Kuzu et al. also showed insignificant differences in the handwashing compliance between physicians and nurses [7]. However, some studies also show significant differences in compliance among health personnel [10–12].

In the scrubbing procedure, *scrub the nail and palm using brush side and the skin of hand and arm using sponge side, in both hands* procedure had the lowest level of compliance with mean score in all three groups of health personnel (1.82 ± 1.152 of the maximum score of 4). Mean score achieved in the procedures of *donning the gown* and *donning the gloves* showed the lowest mean score equal to 1.97 ± 0.158 of the maximum score of 2. In the *donning the gown* procedure, the step of *taking the sterile gown by grasping the inner part of the gown especially to choose the neck part* and the step of *unfolding it away from the body* are steps with the lowest mean score. Whereas for the procedure of donning the gloves, the step of *opening the wrapper of the gloves by hands inside the sleeves of the gown* was the step with the lowest mean score.

The purpose of this study is to present the result of our observations regarding the level of compliance of hand hygiene and gowning technique among consultant surgeons, surgical residents, and nurses in Sardjito Hospital which is an academic hospital. However, there are several limitations in this study. Observations were made by surgical resident, and these conditions can cause bias. Guanche et al. showed that internal audit or observation shows that internal audits tend to record better scores [13, 14]. Other than that, since this research is a pilot study, it has limitations on the small number of samples and short research time. Therefore, we need to do larger scale research with a larger number of samples and a longer research period.

## Conclusions

The mean score of each group of health personnel in each section showed good results. The highest total mean score was achieved by consultant surgeons, however, comparison of hand hygiene and gowning procedure performance between groups of health personnel did not show significant differences. Larger scale research with a larger number of samples, longer research period, and better observer bias control is needed to develop this pilot study.

## Abbreviations

HAI: Healthcare-associated infections
SSI: Surgical site infection
